# FGF7 peptide (FGF7p) mimetic mitigates bladder urothelial injury from cyclophosphamide

**DOI:** 10.14814/phy2.15241

**Published:** 2022-04-07

**Authors:** Sridhar Tatarao Narla, Lori Rice, David Ostrov, Steven G. Swarts, Dietmar W. Siemann, Daniel Scott Bushnell, Jacqueline G. Holden, Joanne Lindsey Duara, Carlton Matthew Bates

**Affiliations:** ^1^ 12317 Division of Nephrology Department of Pediatrics University of Pittsburgh School of Medicine Pittsburgh Pennsylvania USA; ^2^ Department of Radiation Oncology College of Medicine University of Florida Gainesville Florida USA; ^3^ 12233 Department of Pathology, Immunology and Laboratory Medicine University of Florida College of Medicine Gainesville Florida USA; ^4^ 12317 Division of Neonatology Department of Pediatrics University of Pittsburgh School of Medicine Pittsburgh Pennsylvania USA; ^5^ Division of Nephrology UPMC Children’s Hospital of Pittsburgh Pittsburgh Pennsylvania USA

**Keywords:** bladder, cyclophosphamide, FGF7p, fibroblast growth factor 7 peptide, urothelium

## Abstract

Although full‐length fibroblast growth factor 7 (FGF7) blocks cyclophosphamide‐induced urothelial apoptosis in mice, limitations include high production costs because of its large size. We previously identified a small peptide derived from FGF2 that mitigated acute radiation syndrome as well as full‐length FGF2. Based on the sequence of the FGF2 peptide, we synthesized a corresponding 19 amino acid FGF7 peptide (FGF7p). Our objectives were to determine if systemic FGF7p triggered the downstream targets and protected against cyclophosphamide bladder injury similar to full‐length FGF7. We administered FGF7p or vehicle subcutaneously (SQ) to mice subjected to no injury or intraperitoneal (IP) cyclophosphamide and harvested bladders 1 day after injury. We then performed hematoxylin and eosin, TUNEL and immunofluorescence (IF) staining. In uninjured mice, a 20 mg/kg threshold FGF7p dose induced expression of phosphorylated (activated) FRS2α (pFRS2α), and pAKT in urothelium (consistent with cytoprotective effects of FGF7). We then gave FGF7p (20 mg/kg) or vehicle at 72 and 48 h prior to cyclophosphamide. One day after injury, TUNEL staining revealed many more apoptotic urothelial cells with vehicle treatment versus FGF7p treatment. IF for pAKT and readouts of two anti‐apoptotic AKT targets (BAD and mTORC1) revealed minimal staining with vehicle treatment, but strong urothelial expression for all markers with FGF7p treatment. In conclusion, FGF7p appears to block bladder urothelial apoptosis via AKT and its targets, similar to FGF7. FGF7p is much more inexpensive to make and has a longer shelf life and higher purity than FGF7.

## Graphical abstract

We identified a small peptide derived from full‐length fibroblast growth factor 7 (FGF7) that we named FGF7 peptide (FGF7p). Similar to full‐length FGF7, systemic FGF7p drove widespread pFRS2alpha and pAKT staining and limited pERK expression and proliferation in uninjured mouse bladder urothelium. We also noted that pre‐treatment with FGF7p protected against cyclophosphamide‐induced bladder urothelial injury, likely via AKT targets known to be anti‐apoptotic, again similar to full length FGF7.

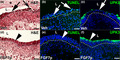

## 1 INTRODUCTION

Hemorrhagic cystitis occurs in up to 35% of patients exposed to cyclophosphamide (Haldar et al., 2014; Korkmaz et al., 2007; Nicol, 2002). Cyclophosphamide is metabolized to acrolein, which is filtered by the kidneys into the urine, where it can injure bladder urothelium, characterized by an initial necrotic loss of lumenal superficial cells followed by apoptosis of intermediate and basal cells (Narla et al., 2020). Acute injury ranges in severity from microscopic hematuria and/or pain with urination to severe hemorrhage. Chronic complications can include bladder fibrosis, contractures, and higher‐risk urothelial cancer (Baker et al., 1987; Kaldor et al., 1995; Shirai, 1993; Vlaovic & Jewett, 1999). One study found that lymphoma survivors given high doses of cyclophosphamide had a 15‐fold higher risk of bladder cancer (Travis et al., 1995). Hemorrhagic cystitis and the chronic complications continue to arise even with current therapies, including sodium‐2‐mercaptoethanesulfonate that binds acrolein or hydration that dilutes urinary acrolein, respectively (Korkmaz et al., 2007; Matz & Hsieh, 2017; Moghe et al., 2015).

In 1997, a study showed that administration of recombinant human fibroblast growth factor 7 (rhFGF7) before giving cyclophosphamide, improved urothelial histology versus vehicle in rats (although the reasons for improvements were not identified) (Ulich et al., 1997). Our group later found that one SQ dose of FGF7 given 24 h prior to IP cyclophosphamide in mice blocked urothelial cell apoptosis, leading to faster and higher fidelity repair versus vehicle treatment (Narla et al., 2020). The FGF7 anti‐apoptotic effects were preceded by urothelial phosphorylation (activation) of FRS2α, likely indicating activation of urothelial fibroblast growth factor receptor 2 (FGFR2), the receptor for FGF7 (Narla et al., 2020); shortly thereafter, FGF7‐treated mice had strong urothelial pAKT staining that correlated with the onset of cytoprotection (Narla et al., 2020) (and AKT activation is well known to block apoptosis Song et al., 2005). A more recent study from our group confirmed the importance of AKT signaling downstream of FGF7 in that co‐administration of an AKT antagonist with full‐length FGF7 blocked the latter's cytoprotective effects against cyclophosphamide (Narla et al., 2021). Furthermore, full‐length FGF7 induced phosphorylation of BCL2‐associated agonist of cell death (pBAD) in urothelium and preserved mammalian target of rapamycin complex 1 (mTORC1) activity (marked by urothelial phospho‐p70S6K [pS6] expression) after cyclophosphamide; AKT is known to act directly on BAD and mTORC1 to block apoptosis (Bui et al., 2018; Igney & Krammer, 2002).

Although FGF7 is a promising therapy to mitigate urothelial injury after cyclophosphamide, its relatively large size requires that it be synthesized by recombinant methods in bacteria, leading to high production and consumer costs, reduced shelf life and decreased purity. Our group previously identified a small peptide derived from full‐length human FGF2, which was as effective as FGF2 in mitigating acute radiation syndrome, but much more inexpensive than FGF2 to produce since it can be directly synthesized (Zhang et al., 2010). The purpose of this study was to generate a corresponding peptide (FGF7p) from FGF7. Once synthesized, we determined the threshold dose and timing to induce urothelial expression of pFRS2α and pAKT in uninjured mice. We then tested the ability of FGF7p to drive cytoprotection, expression of pAKT, pBAD and pS6 expression (the latter two as readouts of anti‐apoptotic AKT targets) in mice given cyclophosphamide.

## 2 MATERIALS AND METHODS

### 2.1 Identification and synthesis of FGF7p

We compared the sequences of full‐length human FGF2 and FGF7 and identified the homologous region of FGF7 that aligned with that of FGF2 peptide. We named this 19 amino acid peptide FGF7 peptide (FGF7p). We then synthesized FGF7p at the University of Pittsburgh Peptide Synthesis Core. Given its relative hydrophobicity, we suspended FGF7p in 2.5% dimethyl sulfoxide (DMSO) (Sigma‐Aldrich, Cat# D2438) and used 2.5% DMSO as the vehicle for all experiments.

### 2.2 Mice

We used 2–3‐month‐old female FVB/NJ mice for all experiments (The Jackson Laboratory). All the proposed mouse experiments were approved by the University of Pittsburgh Institutional Animal Care and Use Committee in accordance with the guidelines of the Association for Assessment and Accreditation of Laboratory Animal Care.

### 2.3 Experimental protocols in mice

To determine the threshold dosing of FGF7p that activated urothelial pFRS2α (readout of FGFR2 activation) and pAKT, we administered three doses of FGF7p (5, 20, and 40 mg/kg SQ) to uninjured mice and assessed staining at 24, 48, and 72 h later. Once we identified the threshold dose that activated pFRS2α and pAKT, we then assessed timing of pERK and Ki‐67 urothelial staining (since we previously found that FGF7 also drives a subset of urothelial cells to proliferate likely in an ERK‐dependent manner Narla et al., 2020). We then assessed the ability of FGF7p to block urothelial apoptosis by administering 150mg/kg IP injections of cyclophosphamide (Sigma‐Aldrich, Cat# C7397) dissolved in PBS. All groups of mice included *N *= 3.

### 2.4 Histology, immunofluorescence, and TUNEL assays

We isolated and fixed bladders in 4% paraformaldehyde, processed and embedded tissues in paraffin, and serially sectioned bladders at 6 µm. For general histology, we stained with hematoxylin and eosin (H&E). For immunofluorescence (IF), we dewaxed the paraffin‐embedded sections and subjected them to antigen retrieval in a pressure cooker for 15 min in Tris‐EDTA pH 9.0 buffer. We then blocked with normal donkey serum for 1 h at room temperature (RT). We then incubated sections overnight at 4°C with the following primary antibodies: Uroplakin 3a (UPK3, superficial cell and intermediate cell subset marker) at 1:200 (Santa Cruz Biotechnology, Cat# sc‐33570, RRID:AB_2213486), pAKT at 1:100 (Cell Signaling Technology, Cat# 4060, RRID:AB_2315049), pERK at 1:50 (Cell Signaling Technology, Cat# 9100, RRID:AB_330741), marker of proliferation Ki‐67 (Ki67) at 1:200 (R&D Systems Cat# AF7649, RRID:AB_2687500), pBAD at 1:100 (Abcam, Cat# AB28824, RRID:AB_725616) and p‐p70S6K (pS6) (Cell Signaling Technology, Cat#4858S, RRID:AB_916156). After washing in PBS, we incubated slides with the following secondary antibodies: Alexa Fluor 594 (Thermo Fisher, Cat# A‐21207, RRID:AB_141637), Alexa Fluor 488 (Thermo Fisher, Cat# A‐21202, RRID:AB_141607), all at 1:500 for 2 h at RT, followed by washes. We stained nuclei with 4′6′‐diamidino‐2‐phenylindole (DAPI) (Sigma Aldrich, Cat# D1306). To assay for apoptosis, we performed TUNEL assays with ApopTag Plus in situ Apoptosis Fluorescein Detection kit (EMD Millipore, Cat# S7111) according to the manufacturer's protocol. We imaged slides with a Leica DM2500 microscope (Leica Microsystems) or a Zeiss LSM 710 confocal microscope (Carl Zeiss) (individual optical sections). Images for the both microscopes were obtained at RT with a 20X objective, except for the images of Ki67 and pERK staining, which were captured with a 40X objective on the confocal microscope. Images for vehicle and treatment groups were obtained at the same settings. All fluorescent images were acquired with the same exposure time. Any subsequent adjustments to brightness and/or contrast on the images were identical for vehicle and treatment groups.

## 3 RESULTS

### 3.1 Identification of FGF7p

**FIGURE 1:**
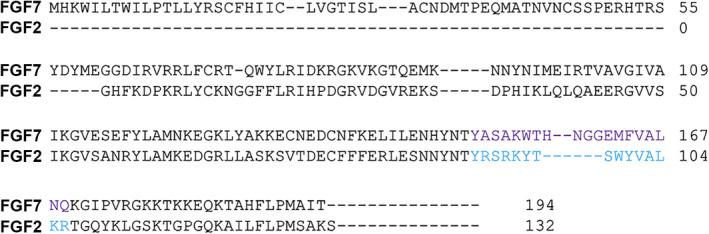
Sequence of FGF7p. Sequence alignment of FGF7 with FGF2 showing regions corresponding to and FGF7 peptide (purple font) and FGF2 peptide (blue font)

We aligned the amino acid sequences of human FGF2 and FGF7 and identified the region of FGF7 that corresponded to the sequence of the FGF2 peptide mimetic and named the region FGF7 peptide (FGF7p). Both human FGF7 and FGF2 peptide mimetics differ from the corresponding mouse peptides by one amino acid. The 19 amino acid sequence of FGF7p is YASAKWTHNGGEMFVALNQ (Figure 1). Given that the human FGF2 peptide is known to activate its receptor in mice (Zhang et al., 2010), we suspected that human FGF7p would interact with and activate its murine receptor, FGFR2.

### 3.2 FGF7p drives downstream signaling in urothelium (pFRS2α, pAKT, pERK, and Ki‐67) similar to FGF7

**FIGURE 2:**
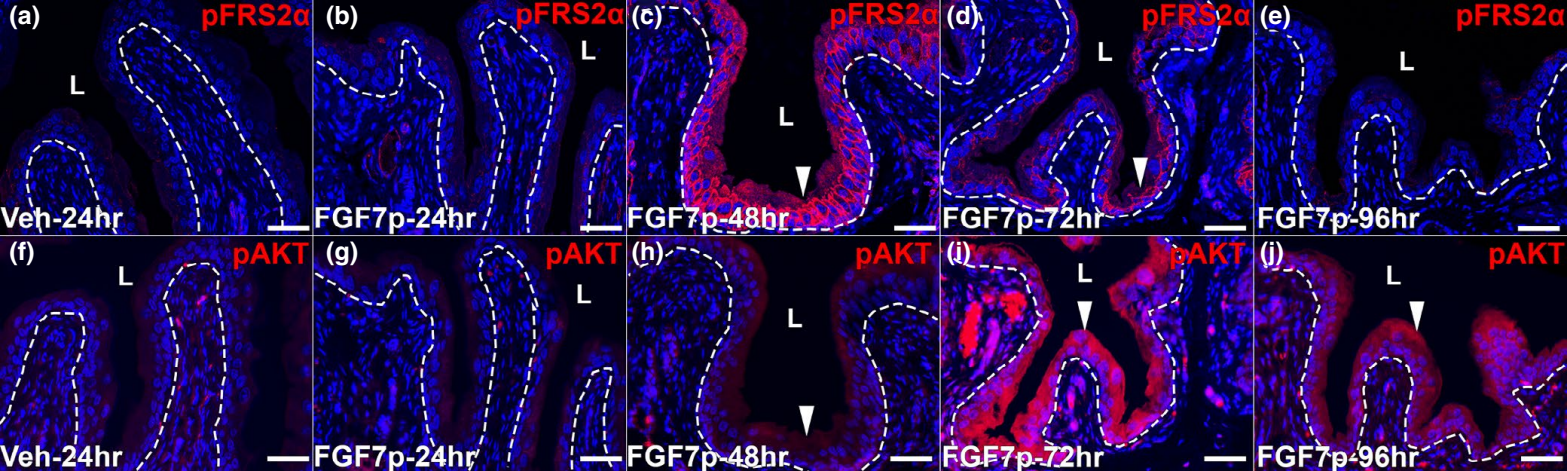
Representative images showing the temporal expression of pFRS2α and pAKT after administration of FGF7p. (a) IF for pFRS2α (red) shows no urothelial staining in mice 24 hours (h) after receiving vehicle (Veh). (b–e) IF for pFRS2α (red) in mice receiving FGF7p reveals no staining at 24 h (b), robust urothelial expression at 48 h (c, arrowhead), reduced staining at 72 h (d, arrowhead) and no expression at 96 h (e) after dosing. (f) IF for pAKT (red) shows no urothelial staining in mice 24 h after receiving vehicle. (g–j) IF for pAKT (red) in mice receiving FGF7p shows no urothelial expression at 24 h (g), light staining at 48 h (h, arrowhead), robust expression at 72 h (i, arrowhead), and reduced staining at 96 h (j, arrowhead). Blue = DAPI. Scale bars (a–j) = 50 µm; Dashed line = Urothelial border with stroma; L, lumen

**FIGURE 3:**
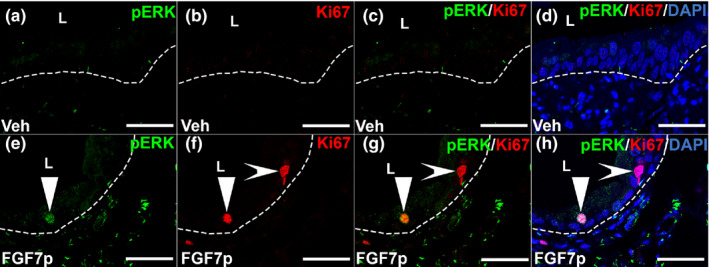
Representative images of pERK and Ki67 staining in uninjured mice 96 h after FGF7p. (a–h) Co‐IF for pERK (green) and Ki67 (red) in vehicle treated (Veh) (a–d) and FGF7p‐treated mice (FGF7p) (e–h). (a–d) Vehicle‐treated mice had no pERK^+^ or Ki67^+^ urothelial cells in the green channel (a), the red channel (b), merged images without DAPI (c) or merged images with DAPI (d). E‐H. FGF7p‐treated mice had subsets of urothelial cells that were pERK^+^ on the green channel (e, arrowhead) and Ki67^+^ cells some of which appeared to also express pERK (f, arrowhead) and others that did not (f, concave arrowhead). Merged images without DAPI (g) and with DAPI (h) confirmed presence of pERK^+^/Ki67^+^ cells (arrowheads) and others that were only Ki67^+^ (concave arrowheads). Scale bars (a–h) = 50 µm; Dashed line = Urothelial border with stroma; L, lumen

We sought to determine if FGF7p could activate FGFR2 urothelial signaling in a manner similar to full‐length FGF7. First, we tested whether FGF7p, like FGF7, could drive pFRS2α expression in the urothelium of uninjured mice, which would be a readout of FGFR2 activation in the urothelium. We administered 5, 20, or 40 mg/kg of FGF7p (vs. 2% DMSO vehicle) SQ and assessed pFRS2α staining at 24, 48, 72, and 96 h later. Mice had no apparent adverse local or systemic side effects from FGF7p or vehicle injections. We observed no urothelial pFRS2α expression with vehicle or with 5 mg/kg of FGF7p at any time point (24‐h vehicle is shown in Figure 2a, and the rest are not shown). At the 20 mg/kg dose, we observed no pFRS2α staining at 24 h, robust expression throughout the urothelium at 48 h, diminishing staining at 72 h and no expression at 96 h later (Figure 2b–e). We saw a similar staining pattern for mice given 40 mg/kg SQ FGF7p (not shown). We then assessed pAKT staining and similar to pFRS2α, we detected no urothelial pAKT staining with vehicle or the 5 mg/kg SQ dose of FGF7p (24‐h vehicle is shown in Figure 2f, and the rest are not shown). The mice given 20 mg/kg of FGF7p had no pAKT expression at 24 h, light urothelial staining at 48 h, robust urothelial expression at 72 h and diminishing signal at 96 h after dosing (Figure 2g–j). We observed similar staining patterns with the 40 mg/kg dose (not shown). We then assessed pERK and Ki67 staining using the same FGF7p dosing strategy. We observed no urothelial staining for either marker with vehicle or 5 mg/kg FGF7p (96‐h vehicle is shown in Figure 3a–d, and the rest are not shown). At the 20 mg/kg dose, we observed no urothelial pERK or Ki67 at 24, 48, or 72 h after FGF7p (not shown); by 96 h we observed small subsets of intermediate cells that were pERK^+^/Ki67^+^ and others that were only Ki67^+^ (Figure 3e–h), similar to what we have seen with full‐length FGF7 treatment (Narla et al., 2020). Thus, similar to full‐length FGF7, FGF7p treatment led to urothelial activation of FRS2α followed by pAKT staining throughout the urothelium and pERK staining in intermediate urothelial cell subsets that were also Ki67^+^ (i.e., proliferating) (as well as pERK^−^/Ki67^+^ cells) (Narla et al., 2020). One difference is that we had to use a higher dose of FGF7p (20 mg/kg) to drive urothelial signaling than full‐length FGF7 (5 mg/kg).

### 3.3 FGF7p drives urothelial cytoprotection against cyclophosphamide, likely via AKT, similar to FGF7

**FIGURE 4:**
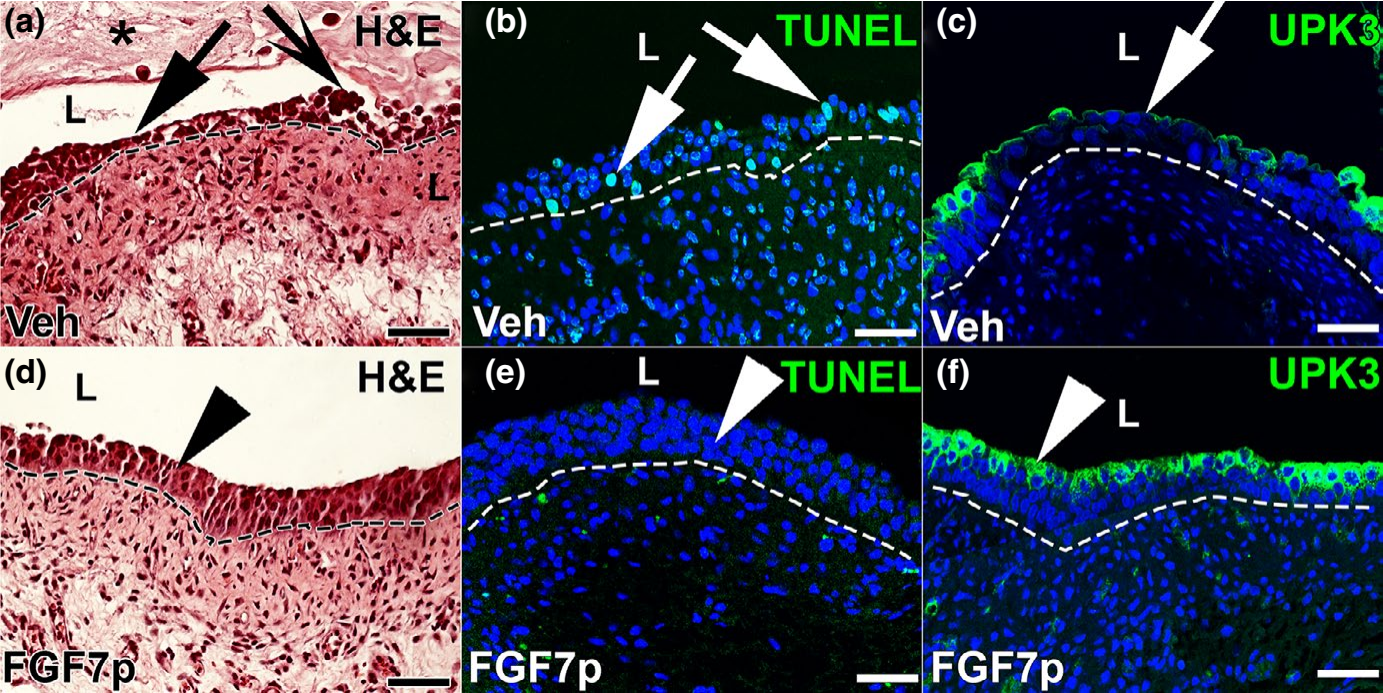
Representative images showing urothelial cytoprotection from FGF7p in cyclophosphamide‐injured mice 24 h after injury. (a–f) Images from injured vehicle‐treated mice (Veh) (a–c) and injured FGF7p‐treated mice (FGF7p) (d–f) showing injury patterns. (a) H&E image shows that vehicle‐treated mice have significant urothelial injury with denuding (arrow) and sloughing cells (concave arrow), with significant cellular debris in the lumen (asterisk). (b) TUNEL staining (green) reveals many TUNEL^+^ apoptotic urothelial cells (arrows) in vehicle‐treated mice. (c) IF for UPK3 (green) shows regional losses of urothelial staining (arrow) in vehicle‐treated mice. (d) H&E image shows that FGF7p‐treated mice have largely intact urothelial cells (arrowhead). (e) TUNEL staining (green) reveals virtually no urothelial apoptosis in FGF7p‐treated mice (arrowhead). (f) IF for UPK3 (green) shows relatively intact expression in FGF7p‐treated mice (arrowhead), consistent with preservation of urothelial cell layers. Blue (b–e, e, f) = DAPI. Scale bars (a–f) = 50 µm. Dashed line = Urothelial border with stroma; L, lumen

We next assessed whether FGF7p was able to block cyclophosphamide‐induced urothelial apoptosis similar to full‐length FGF7. To that end, we gave mice vehicle or 20mg/kg of FGF7p at 72 and 48 h prior to cyclophosphamide at 150 mg/kg IP and then collected bladders 24 h after injury. H&E staining sections showed that vehicle‐treated and injured mice had marked urothelial denuding and sloughing along with cellular debris in the lumen (Figure 4a); in contrast, FGF7p‐treated and injured mice had relative preservation of urothelial cells (Figure 4d). TUNEL staining showed that vehicle‐treated mice had many apoptotic urothelial cells (Figure 4b), while FGF7p‐treated mice had almost no urothelial apoptosis (Figure 4e) after cyclophosphamide. Finally, IF for UPK3 showed regions of absent urothelial staining in the vehicle groups (Figure 4c), while expression in the FGF7p group was largely intact (Figure 4f) after injury. Thus, FGF7p appears to confer a similar degree of urothelial cytoprotection from cyclophosphamide as full‐length FGF7 (Narla et al., 2020).

**FIGURE 5:**
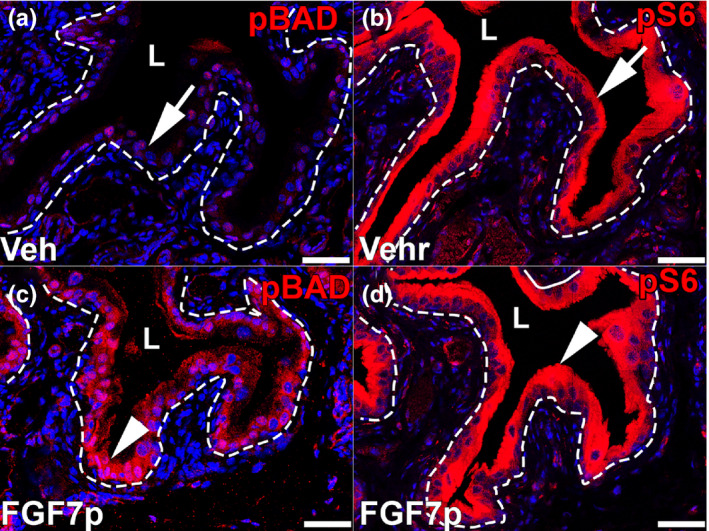
Representative images showing effects of FGF7p on AKT targets, BAD, and mTORC1, 72 h after treatment in uninjured mice. (a, b) In uninjured mice, IF for pBAD (red), a readout of BAD suppression, shows minimal cytoplasmic urothelial staining with vehicle (Veh) treatment (a, arrow), but robust cytoplasmic signal with FGF7p treatment (b, arrowhead). (c, d) In uninjured mice, IF for pS6 (red), a readout of mTORC1 activity, shows cytoplasmic urothelial staining that appears relatively equivalent with vehicle treatment (c, arrow) and FGF7p treatment (d, arrowhead). Blue = DAPI. Scale bars (a–d) = 50 µm; Dashed line = Urothelial border with stroma; L, lumen

We then assessed whether the FGF7p‐induced cytoprotection correlated with activation of AKT and two of its downstream targets known to affect apoptosis, BCL2 associated agonist of cell death (BAD) and mammalian target of rapamycin complex 1 (mTORC1). In addition to phosphorylating AKT (Narla et al., 2020), we previously found that full‐length FGF7 leads to phosphorylation of BAD and prevents cyclophosphamide‐induced loss of pS6 urothelial staining (readout of mTORC1 signaling), both of which should block apoptosis (Narla et al., 2021). We now assessed if FGF7p treatment mimicked that of full‐length FGF7 in regard to AKT, and these latter two targets. Complementing our earlier assays assessing pAKT in uninjured mice, we administered a single 20 mg/kg dose of FGF7p or vehicle and assessed pBAD and pS6 72 h later (with no cyclophosphamide). Similar to what we observed with full‐length FGF7 (Narla et al., 2021), we noted induction of cytoplasmic urothelial pBAD staining in FGF7p‐treated mice versus vehicle and robust baseline cytoplasmic urothelial pS6 expression in both vehicle and FGF7p‐treated mice without injury (Figure 5).

We next assessed FGF7p‐induced changes in pAKT, pBAD, and pS6 staining in the context of injury.

**FIGURE 6:**
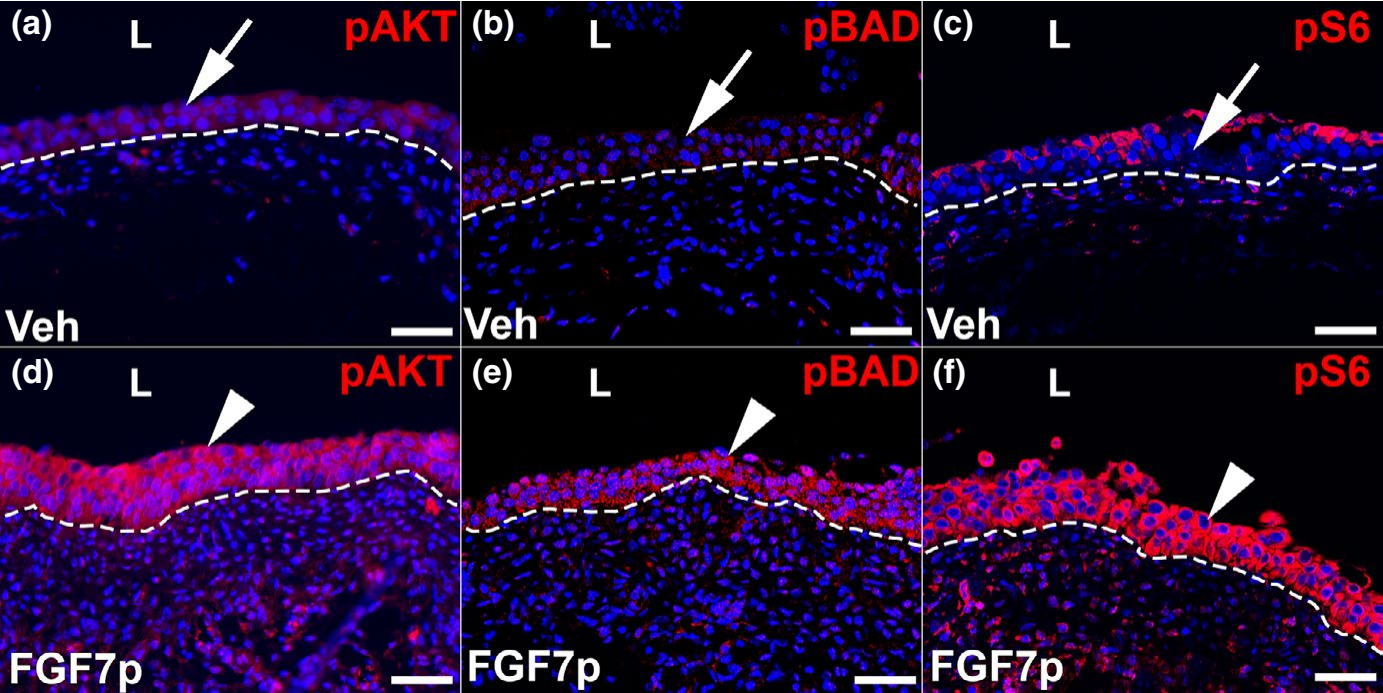
Representative images showing activation of AKT and readouts of its downstream targets after FGF7p treatment in cyclophosphamide‐injured mice 24 h after injury. (a–f) Images from vehicle‐treated mice (Veh) (a–c) and injured FGF7p‐treated mice (FGF7p) (d–f) revealing expression of pAKT and readouts of its targets (pBAD and pS6). (a) IF for pAKT (red) shows limited urothelial pAKT staining (arrow) in vehicle‐treated mice. (b) IF for pBAD (red) reveals limited cytoplasmic urothelial expression (arrow) In injured mice treated with vehicle. (c) IF for pS6 (red) shows diminished cytoplasmic urothelial staining (arrow) in injured mice treated with vehicle. (d) IF for pAKT (red) shows induction of staining in the urothelium (arrowhead) in FGF7p‐treated mice. (e) IF for pBAD (red) also shows induction of expression (arrowhead) in injured mice given FGF7p. (f) IF for pS6 (red) reveals preservation of cytoplasmic urothelial expression (arrowhead) in injured mice given FGF7p. Blue = DAPI; Scale bars (a–f) = 50 µm; Dashed line = Urothelial border with stroma; L, lumen

Thus, we used the same dossing strategy as above (two doses of FGF7p followed by cyclophosphamide and then harvest 24 h later) and performed IF studies. As expected, vehicle‐treated and injured mice had minimal urothelial pAKT staining (Figure 6a), while FGF7p‐treated and injured mice had significant induction of urothelial pAKT expression (Figure 6d). Similarly, vehicle‐treated mice had virtually no cytoplasmic urothelial pBAD staining (Figure 6b), whereas FGF7p‐treated mice had significant induction of expression (Figure 6e). Finally, vehicle‐injured mice had significant loss of pS6 urothelial expression (Figure 6c), whereas FGF7p mice had preservation of cytoplasmic urothelial staining (Figure 6f). Thus, similar to full‐length FGF7, FGF7p treatment led to urothelial cytoprotection against cyclophosphamide, which correlated with activation of AKT and modulation of downstream AKT targets that should block apoptosis.

## 4 DISCUSSION

Our study identified a 19 amino acid peptide derivative from full‐length FGF7 that we called FGF7p. FGF7p is from the region of FGF7 that is equivalent to where the peptide mimetic, FGF2 peptide, is derived from its parent ligand, FGF2 (Zhang et al., 2010). The FGF2 mimetic peptide has been shown to act in a manner similar to full‐length FGF2 in mitigating acute radiation syndrome. Likewise, our report shows that systemic FGF7p acts in a manner similar to full‐length FGF7 (Narla et al., 2020) in apparently activating FGFR2 in the urothelium (noted by pFRS2α staining). Trailing pFRS2α staining, FGF7p treatment led to pAKT expression throughout the urothelium, which correlated with the cytoprotection similar to full‐length FGF7 (Narla et al., 2020). FGF7p also led to pERK staining in a subset of urothelial cells that were Ki67^+^ (as well as cells that were Ki67^+^ only), again mimicking the actions of full‐length FGF7 (Narla et al., 2020). Furthermore, the FGF7p pAKT‐induction was paralleled by increase in pBAD and preservation of pS6 staining, all of which should block apoptosis (similar to what we observed with full‐length FGF7 Narla et al., 2021). Thus the biological activity of FGF7p clearly mimics that of the full‐length FGF7.

As noted, as we have seen with full‐length FGF7 (Narla et al., 2021), the FGF7p‐AKT signaling axis in urothelium appears to modify signaling of at least two AKT targets, BAD and mTORC1. AKT phosphorylates BAD, leading to its redistribution from mitochondria to the cytosol, relieving BAD inhibition of anti‐apoptotic BCL‐X_L_ and BCL2 proteins (Bui et al., 2018; Igney & Krammer, 2002). Moreover, we found that FGF7p induces urothelial pBAD expression, in a similar temporal manner that mimics induction of urothelial pAKT expression (72 h after dosing). AKT also phosphorylates TSC2, thus de‐repressing mTORC1 signaling; mTORC1 then stimulates translation of the anti‐apoptotic protein MCL‐1 and phosphorylates (activates) p70S6K (pS6) that in turn phosphorylates BAD (Ban & Kozar, 2012; Fulda, 2014; Harada et al., 2001; Hosoi et al., 1999; Mills et al., 2008). While we did not observe increased pS6 urothelial staining with FGF7p in uninjured mice, we did note that cyclophosphamide injury alone led to a marked reduction of cytoplasmic urothelial staining, which was prevented by FGF7p (again mimicking what we have seen with full‐length FGF7 Narla et al., 2021). Moreover, cyclophosphamide‐induced urothelial loss of pS6K expression is consistent with a report that acrolein (cyclophosphamide toxic metabolite) led to a reduction in pS6K in cultured endothelial cells starting 4 h after injury (Lemaitre et al., 2011). Thus, the preservation of pS6 expression (mTORC1 activity) also likely contributes to the cytoprotective effects of FGF7p in the urothelium. Thus, FGF7p‐induced activation of pBAD and preservation of pS6 urothelial staining likely lead to urothelial cytoprotection against cyclophosphamide, mimicking the actions of full‐length FGF7 (Narla et al., 2021).

On balance, use of FGF7p to mitigate bladder injury from cyclophosphamide has many potential advantages over full‐length FGF7. One minor disadvantage is that the threshold dose of FGF7p that leads to pFRS2α and pAKT expression in the bladder is higher than FGF7 (20 vs. 5 mg/kg, respectively). The FGF2 mimetic also has a higher threshold dose driving activity than FGF2 in acute radiation syndrome (Zhang et al., 2010). The biggest advantage of FGF7p over FGF7 is significant production (and potentially consumer) costs. Although full‐length rhFGF7 costs ~$5200 for 1 mg (R&D Systems, Minneapolis, MN, Cat# 251‐KG‐010), we can synthesize FGF7p in the University of Pittsburgh Peptide Synthesis core for ~$24 for 1 mg (<0.5% of the commercial cost of rhFG7). If FGF7p were mass produced, it would lead to more cost savings. Lyophilized FGF7p also has a longer shelf life (5–10 years) than rhFGF7 (1 year‐R&D Systems) and direct synthesis allows for higher purity than recombinant production. Although systemic use of FGF7 appears safe, particularly as a “one‐off” therapy, recurrent systemic FGF7 dosing could lead to unwanted side effects. Future studies could assess long‐term off‐target effects of systemic FGF7p in mice; we could obtain blood for metabolic panels (e.g., serum electrolytes, serum creatinine, blood urea nitrogen, liver function tests, amylase, lipase, etc.), complete blood counts (to look for signs of cancer) and full necropsies in which we would harvest, weigh, section and perform histological staining on multiple organs. Notably, we have harvested and performed histological stains on many organs (heart, lung, liver, kidney, brain, and bladder) in mice 6 months after a single dose of full‐length FGF7 and have seen no abnormalities relative to vehicle‐treated mice (*N* = 4 per group) (unpublished data). A potential solution to any unwanted side effects encountered could be to directly instill FGF7 transurethrally into the bladder; however, full‐length FGF7 is likely too large to taken up by urothelial cells (data from a rodent study also indirectly support a non‐trophic effect of local KGF on urothelium Ulich et al., 1997). Conversely, the relatively small size of FGF7p might allow it to traverse the urothelial barrier and work if instilled directly into the bladder through the urethra. While beyond the scope of this study, FGF7p might block other types of bladder urothelial injury such as radiation injury or neuropathic or could protect against other types of epithelial injury for which FGF7 is beneficial (i.e., oral, retinal, alveolar, or intestinal epithelium Dorr et al., 2002; Farrell et al., 1998, 1999, 2002; Hu et al., 2018; Khan et al., 1997; Takeoka et al., 1997; Wu et al., 1998).

## CONFLICT OF INTEREST

The authors have no conflicts to declare.

## AUTHOR CONTRIBUTIONS

Sridhar Narla: Study design; conducting experiments; analysis; writing and editing. Lori Rice: Conceptualization; analysis; writing, reviewing and editing. David Ostrov: Conceptualization; analysis; writing, reviewing and editing. Steven Swarts: Validation; writing, reviewing and editing. Dietmar Siemann: Validation; writing, reviewing and editing. Daniel Bushnell: Conducting experiments; writing. Jacqueline Holden: Conducting experiments; writing. Joanne Duara: Conducting experiments; writing. Carlton Bates: Conceptualization; supervision; analysis writing, reviewing, editing.
